# ﻿A new species of *Sedum* (Crassulaceae) from Mount Danxia in Guangdong, China

**DOI:** 10.3897/phytokeys.221.97495

**Published:** 2023-03-10

**Authors:** Yan-Shuang Huang, Kai-Kai Meng, Yuan-Yuan Sun, Zai-Xiong Chen, Qiang Fan

**Affiliations:** 1 State Key Laboratory of Biocontrol and Guangdong Provincial Key Laboratory of Plant Resources, School of Life Sciences, Sun Yat-sen University, Guangzhou 510275, China; 2 School of Ecology, Sun Yat-sen University, Shenzhen 518107, China; 3 Administrative Commission of Danxiashan National Park, Shaoguan 512300, China

**Keywords:** Danxia landscape, morphology, nrITS, *Sedum* sect. *Sedum*

## Abstract

*Sedumjinglanii*, a new species of Crassulaceae from Mount Danxia in Guangdong, China, is described and illustrated. Phylogenetic analysis based on the internal transcribed spacer (ITS) region of *nr*DNA suggests that the new species belongs to S.sect.Sedum sensu Fu and Ohba (2001) in the “Flora of China”, and is sister to a clade comprising *S.alfredi* and *S.emarginatum* with high support values (SH-aLRT = 84, UFBS = 95) but is distantly related to *S.baileyi*. The new species is morphologically similar to *S.alfredi* but it can be distinguished from the latter in its opposite leaves (vs. alternate leaves), its usually wider leaves (0.4–1.2 cm vs. 0.2–0.6 cm), its usually shorter petals (3.4–4.5 mm vs. 4–6 mm), its shorter nectar scales (0.4–0.5 mm vs. 0.5–1 mm), its shorter carpels (1.5–2.6 mm vs. 4–5 mm), and its shorter styles (0.6–0.9 mm vs. 1–2 mm). The new species can be easily distinguished from *S.emarginatum* which both have opposite leaves by its short, erect or ascending rhizome (vs. long and prostrate rhizome in the latter), shorter petals (3.4–4.5 mm vs. 6–8 mm) and shorter carpels (1.5–2.6 mm vs. 4–5 mm). It can also be easily distinguished from *S.baileyi* by its short, erect or ascending rhizome (vs. long and prostrate rhizome) and its shorter style (0.6–0.9 mm vs. 1–1.5 mm).

## ﻿Introduction

According to Fu and Ohba (2001) in the “Flora of China”, *Sedum* Linnaeus is the most species-rich genus of the family Crassulaceae, comprising about 470 species. However, as presently circumscribed, the genus is highly polyphyletic, and a monophyletic circumscribed genus *Sedum* s.l. would comprise approximately 755 species by inclusion of all 14 genera currently recognized in tribe Sedeae into it (Messerschmid et al. 2020). The genus is distributed in temperate and subtropical environments, and the diversity center is in the Mediterranean Sea, Central America, the Himalayas and East Asia (Stephenson 1994; Thiede and Eggli 2007). In China, 121 *Sedum* species are documented, amongst which 91 species are endemic (Fu and Ohba 2001).

During the past 20 years, about seventeen *Sedum* species have been newly described from China, including *S.hoi* X.F.Jin & B.Y.Ding (Wang et al. 2005), *S.plumbizincicola* X.H.Guo & S.B.Zhou (Wu et al. 2012), *S.fanjingshanense* C.D.Yang & X.Yu Wang (Yang et al. 2012), *S.kuntsunianum* X.F.Jin, S.H.Jin & B.Y.Ding (Jin et al. 2013), *S.tarokoense* H.W.Lin & J.C.Wang (Lu et al. 2013), *S.spiralifolium* D.Q.Wang, D.M.Xie & L.Q.Huang (Xie et al. 2014), *S.peltatum* M.L.Chen & X.H.Cao (Chen et al. 2017), *S.kwanwuense* H.W.Lin, J.C.Wang & C.T.Lu and *S.taiwanalpinum* H.W.Lin, J.C.Wang & C.T.Lu (Lu et al. 2019), *S.ichangense* Y.B.Wang (Wang and Xiong 2019), *S.lipingense* R.B.Zhang, D.Tan & R.X.Wei (Zhang et al. 2019), *S.nanlingense* Yan Liu & C.Y.Zou (Zou et al. 2020), *S.cirenianum* S.S.Ying, *S.shaoakouense* S.S.Ying and *S.shengkuangense* S.S.Ying (Ying 2022a), and *S.parviflorum* S.S.Ying and *S.tachingshuianum* S.S.Ying (Ying 2022b).

Molecular data unambiguously demonstrate the polyphyletic nature of *Sedum* with its species placed in four major crown clades of the crassulacean tree, for example, *Acre*, *Aeonium*, *Leucosedum*, and *Sempervivum*. There is no agreement between specialists regarding the infrageneric structure of *Sedum* (reviewed in Nikulin et al. 2016). According to Fu and Ohba (2001), Chinese *Sedum* are divided into three sections, including S.sect.Sedum, S.sect.Oreades (Fröderström) K.T. Fu, and S.sect.Filipes (Fröderström) S.H. Fu. S.sect.Sedum is distinguishable from the two latter sections by the adaxially gibbous carpels and follicles, while S.sect.Oreades differs from S.sect.Filipes in the spurred leaf base and yellow petals (vs. spurless leaf base and white or reddish-purple petals) (Fu and Ohba 2001). During our investigations in Danxiashan National Park, Guangdong Province, China, an unknown *Sedum* species with opposite leaves was collected. After several years of field observations, comprehensive literature studies and molecular analysis, we confirmed that it was a new species and it is described and illustrated here.

## ﻿Materials and method

Field investigations and observations were conducted during the ﬂowering and fruiting periods of the putative new species. We obtained morphological data of this putative species by measurements based on 6–8 living samples. Mean values of these statistical data were calculated and then were compared with six other related species (Table 2). The specimens were collected in Danxiashan National Park, Renhua County, Guangdong Province, China. Voucher specimens were deposited in the herbarium of Sun Yat-sen University (**SYS**).

Two representative individuals from different populations were selected for further molecular experiments, one from Bazhai of Mount Danxia (*Y. S. Huang 21040301*) and another one from Yanyan of Mount Danxia (*Q. Fan et al. DNPC 2873*). Fresh leaves of the two individuals were collected and stored with silica gel in zip-lock plastic bags until use. Total DNA was extracted using the modified CTAB method (Doyle and Doyle 1987). The region of the partial internal transcribed spacer 1, 5.8S ribosomal RNA gene and partial internal transcribed spacer 2 was amplified using previously reported primers ITS1 and ITS4 (White et al. 1990). PCR amplifications were performed following Huang et al. (2021).

In order to explore the phylogenetic position of the putative new species in *Sedum*, ITS sequences of 56 accessions representing 46 *Sedum* taxa and three outgroup species (*Aeoniumlancerottense*, *Aeoniumviscatum*, and *Greenoviaaizoon*) were downloaded from the Genbank public database at the National Center for Biotechnology Information (NCBI) (Table 1). The sequences were aligned using ClustalW 1.8 (Thompson et al. 1994) and then adjusted manually. Besides, to improve the credibility, we also aligned the sequences using MAFFT v.7.402 (Katoh and Standley 2013), and the alignments generated from the two methods were consistent. The best-fit nucleotide substitution model was determined by ModelFinder (Kalyaanamoorthy et al. 2017). Based on the maximum likelihood (ML) method, the phylogenetic tree was constructed using IQ-Tree v. 2.0.3 (Nguyen et al. 2015) by executing 5,000 replicates of SH approximate likelihood ratio test (SH-aLRT) and ultrafast bootstrap (UFBS) (Hoang et al. 2018). Finally, the tree file was visualized by the online tool of Interactive Tree Of Life (iTOL) v5 (Letunic and Bork 2021).

**Table 1.** Taxa, voucher information, and GenBank accession numbers of the sequences used in this study.

Taxon	Voucher	Accession numbers	References
SedumSect.Oreades			
*S.oreades*	*Rao 090803-03*	KF113733	Zhang et al. 2014
*S.trullipetalum*	*Miyamoto et al. 9420132*	AB088630	Mayuzumi and Ohba 2004
*S.bergeri*	*Ni et al.*	AY352897	Ni et al. unpublished
*S.erici-magnusii*	*Ito 2077*	LC229235	Ito et al. 2017a
SedumSect.Sedum			
*S.jinglanii*	*Huang 21040301*	OP288035	This study
	*Fan et al. DNPC 2873*	OQ162326	This study
*S.actinocarpum*	*Ito 1749*	LC229265	Ito et al. 2017a
*S.alfredi*	*Kokubugata 17190*	AB930259	Ito et al. 2014a
	*Kokubugata 17191*	AB930260	Ito et al. 2014a
	*Kokubugata 17192*	AB930261	Ito et al. 2014a
	*WUK415208*	FJ919953	Wang and Shu unpublished
*S.baileyi*	*LBG0064555*	FJ919935	Wang and Shu unpublished
*S.bulbiferum*	*Ito 416*	LC229234	Ito et al. 2017a
	*130514hs41*	KM111166	Xie et al. 2014
	*130524qz09*	KM111165	Xie et al. 2014
*S.emarginatum*	*130512hs27*	KM111145	Xie et al. 2014
*S.erythrospermum*	*Tsutsumi 1504*	AB906473	Ito et al. 2014b
*S.formosanum*	*Ito 1260*	LC229279	Ito et al. 2017a
*S.hakonense*	*Mayuzumi C00005*	AB088625	Mayuzumi and Ohba 2004
*S.hangzhouense*	*Ito 2604*	LC260130	Ito et al. 2017b
*S.japonicum*	*Kokubugata 16749*	AB906475	Ito et al. 2014b
*S.senanense*	*Ito 2200*	LC229238	Ito et al. 2017a
*S.oryzifolium*	*Ito 2285*	LC229239	Ito et al. 2017a
S.japonicumvar.pumilum	*Ito 2287*	LC229240	Ito et al. 2017a
S.japonicumssp.uniflorum	*Ito 447*	LC229241	Ito et al. 2017a
*S.boninense*	*Ito 2371*	LC229242	Ito et al. 2017a
*S.jiulungshanense*	*Ito 76*	LC229243	Ito et al. 2017a
*S.kiangnanense*	*CMQ1030*	LC229244	Ito et al. 2017a
*S.lineare*	*Mayuzumi C00120*	AB088623	Mayuzumi and Ohba 2004
*S.lungtsuanense*	*Ito 3563*	LC260131	Ito et al. 2017b
*S.makinoi*	*Kokubugata 16730*	AB906476	Ito et al. 2014b
*S.morrisonense*	*Ito 2765*	LC229290	Ito et al. 2017a
*S.multicaule*	*Miyamoto et al. TI9596136*	AB088631	Mayuzumi and Ohba 2004
*S.nagasakianum*	*Ito 2064*	LC229249	Ito et al. 2017a
*S.nokoense*	*Kokubugata 10426*	AB906478	Ito et al. 2014b
*S.oligospermum*	*Ito 74*	LC229250	Ito et al. 2017a
*S.yabeanum*	*Ito 396*	AB906490	Ito et al. 2014b
S.polytrichoidesvar.setouchiense	*Ito 2298*	LC229253	Ito et al. 2017a
*S.polytrichoides*	*CMQ1057*	LC229251	Ito et al. 2017a
*S.rupifragum*	*Ito 2070*	LC229254	Ito et al. 2017a
*S.sarmentosum*	*Ito 978*	LC229255	Ito et al. 2017a
*S.satumense*	*Ito 2295*	LC229256	Ito et al. 2017a
*S.subtile*	*Shimizu 1999*	AB088622	Mayuzumi and Ohba 2004
	*Ito 2259*	LC229257	Ito et al. 2017a
*S.subtile*	*Ito 624*	AB930277	Ito et al. 2014a
*S.taiwanianum*	*Ito 2770*	LC229297	Ito et al. 2017a
*S.tetractinum*	*Ito 3623*	LC260135	Ito et al. 2017b
*S.tianmushanense*	*Ito 355*	LC229261	Ito et al. 2017a
*S.tosaense*	*Kokubugata 16726*	AB906483	Ito et al. 2014b
*S.triactina*	*9596091*	AB088629	Mayuzumi and Ohba 2004
*S.tricarpum*	*Ito 2269*	LC229259	Ito et al. 2017a
S.lipingense ^*^	*ZRB1479*	MN150061	Zhang et al. 2019
S.mexicanum ^*^	*Ito 647*	LC229247	Ito et al. 2017a
S.truncatistigmum ^*^	*Ito 3254*	LC229306	Ito et al. 2017a
S.zentaro-tashiroi ^*^	*Ohba 1998*	AB088619	Mayuzumi and Ohba 2004
Outgroups			
*Aeoniumlancerottense*	*Mort 1518*	AY082143	Mort et al. 2002
*Aeoniumviscatum*	*Mort 1432*	AY082154	Mort et al. 2002
*Greenoviaaizoon*	*Mort 1425*	AY082112	Mort et al. 2002

*Not recorded in Fu and Ohba (2001).

## ﻿Results and discussion

The alignment length of the ITS sequences was 624 bp, amongst which 340 were parsimony-informative. Within the new species, only one variable site was detected, but 40 variable sites were detected between the new species and *S.alfredi* and 40 variable sites were detected between the new species and *S.emarginatum*, indicating that pronounced genetic differentiation existed between the new species and *S.alfredi* as well as *S.emarginatum*. The best-fit nucleotide substitution model was estimated as SYM+I+G4 according to the Bayesian Information Criterion (BIC).

**Figure 1.**
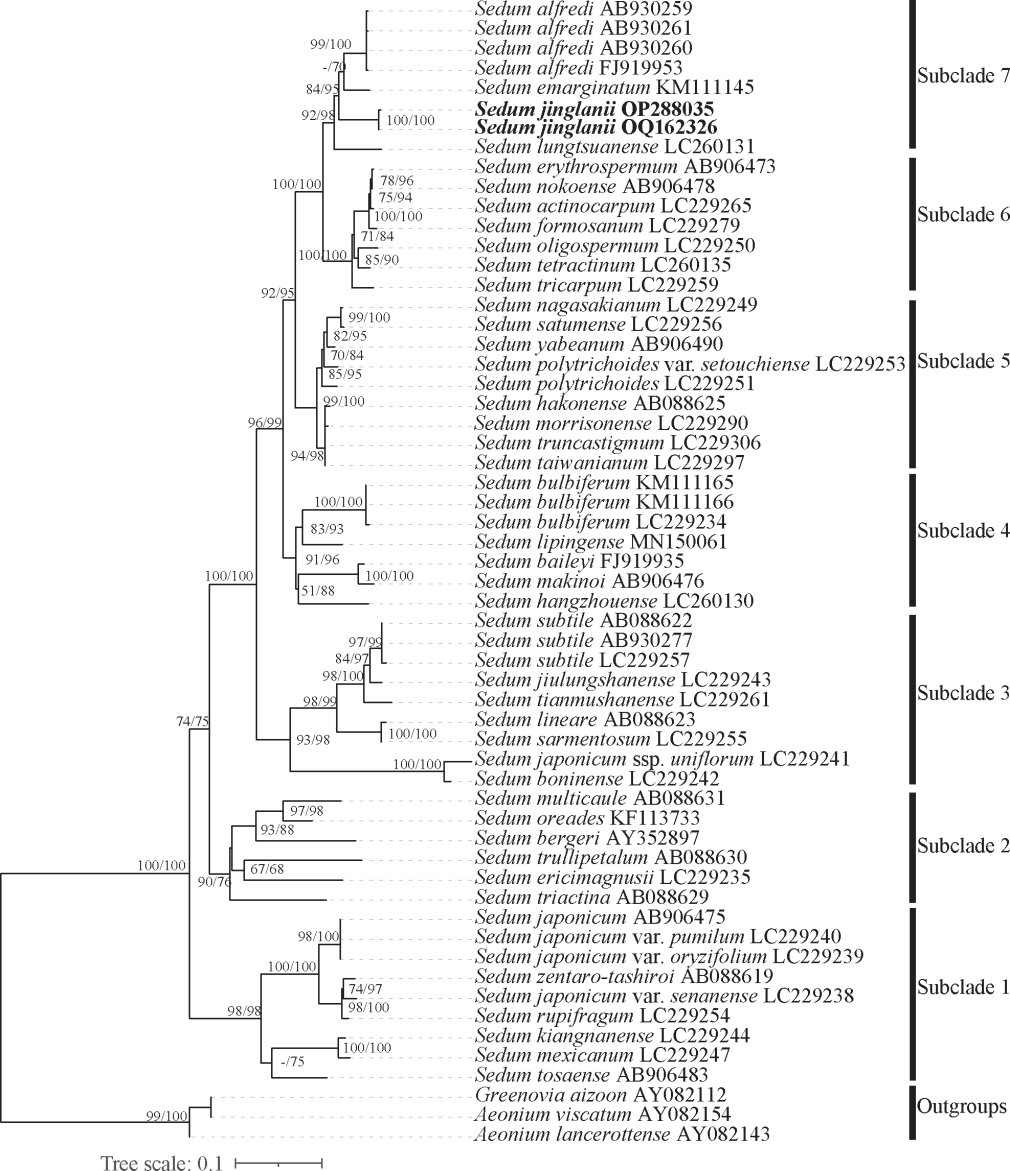
Maximum Likelihood tree based on ITS sequences for Eastern Asian species of Sedumsect.Sedum, four species of S.sect.Oreades, and three outgroups. Numbers near the branches are SH approximate likelihood ratio test (SH-aLRT) and ultrafast bootstrap (UFBS) support values. The new species is highlighted in bold.

As the ML phylogenetic tree shows (Fig. 1), seven subclades were resolved with moderate to high support values. Accessions of the putative new species, *S.alfredi*, *S.emarginatum*, and *S.lungtsuanense* together formed subclade 7 with high support values (SH-aLRT = 92, UFBS = 98), all belonging to S.sect.Sedum sensu Fu and Ohba (2001).

Morphologically, the putative new species is similar to *S.alfredi* from which it can be easily distinguished by its opposite leaves (vs. alternate leaves in the latter). Furthermore, the leaves of the putative new species are usually wider than those of *S.alfredi* (0.4–1.2 mm vs. 0.2–0.6 mm), the petals are usually shorter (3.4–4.5 mm vs. 4–6 mm), the nectar scales shorter (0.4–0.5 mm vs. 0.5–1 mm), the carpels shorter (1.5–2.6 mm vs. 4–5 mm) and the styles shorter (0.6–0.9 mm vs. 1–2 mm) (Table 2). Phylogenetically, the putative new species is closely related to *S.emarginatum*. Although the leaves of both species are opposite, it can be easily distinguished from the latter by its short, erect or ascending rhizome (vs. long and prostrate rhizome), shorter petals (3.4–4.5 mm vs. 6–8 mm) and shorter carpels (1.5–2.6 mm vs. 4–5 mm). The putative new species was distantly related to *S.baileyi* in the phylogenetic tree although both are morphologically similar (Table 2). Also, it can be easily distinguished from the latter by its short, erect or ascending rhizome (vs. long and prostrate rhizome) and its shorter style (0.6–0.9 mm vs. 1–1.5 mm).

**Table 2.** Morphological comparisons between *S.jinglanii*, *S.alfredi*, *S.baileyi*, *S.emarginatum*, *S.kuntsunianum*, *S.makinoi*, and *S.satumense*.

Characters	*S.jinglanii*	*S.alfredi* ^†^	*S.baileyi* ^‡^	*S.emarginatum* ^§^	*S.kuntsunianum* ^|^	*S.makinoi* ^¶^	*S.satumense* ^#^
Leaf blade	Spatulate or obovate	Linear-cuneate, spatulate or obovate	Obovate-spatulate	Spatulate-obovate to broadly obovate	Widely obovate or suborbiculate, spatulate	Obovate or obovate-spatulate	Narrowly obovate or spatulate
Leaf size (cm)	0.8–2.9 × 0.4–1.2	1.2–3.0 × 0.2–0.6	1–2.5 × 0.6–0.8	1–2.5 × 0.5–1.2	1.4–2.0 × 0.9–1.5	1–2 × 0.6–0.8	1.0–2.2 × 0.6–0.9
Phyllotaxy	Opposite	Alternate	Opposite	Opposite	Opposite, or rarely alternate at base	Opposite	Opposite
Rhizome	Short, erect or ascending	Short, erect or ascending	Long, prostrate	Long, prostrate	Absent	Short, erect or ascending	Short, erect or ascending
Sepal length (mm)	2–3.1	2–5	1.5–2.5	2–5	5–9	2–3	6–7
Petal length (mm)	3.4–4.5	4–6	4–5	6–8	7–8	4–5	7–8
Stamen length (antepetalous) (mm)	2.2–2.6	2.5–3.5	2–3	3–4	ca. 5	2.5–3.2	–
Stamen length (antesepalous) (mm)	3.2–3.3	3.8–4.5	3–4	4–5	ca. 6	2.8–4.5	–
Nectar scale length (mm)	0.4–0.5	0.5–1	0.4–0.6	0.6–0.8	ca. 0.5	0.5–0.7	ca. 0.5
Carpel length (mm)	1.5–2.6	4–5	2–3	4–5	ca. 5	4–5	–
Style length (mm)	0.6–0.9	1–2	1–1.5	1.5–2	ca. 1	1–2	1.0–1.5

**^†^**,**^‡^**,**^§^**,**^¶^** Based on Fu and Ohba (2001) and own measurements at IBSC and SYS; **^|^**Jin et al. (2013); **^#^**Ohba (2003).

Additionally, four representatives of Sedumsect.Oreades sensu Fu and Ohba (2001) (*S.oreades*, *S.trullipetalum*, *S.bergeri*, and *S.erici-magnusii*) were also included in our analysis. However, these four species were nested within species belonging to S.sect.Sedum sensu Fu and Ohba (2001), thus showing that S.sect.Sedum might not be monophyletic. This result is consistent with previous studies (Nikulin et al. 2016; Zhang et al. 2019; Messerschmid et al. 2020).

Through numerous scientific investigations, more than a dozen new species were found on Mount Danxia in Guangdong in recent years, and most are endemic to it such as *Lespedezadanxiaensis* Q.Fan, W.Y.Zhao & K.W.Jiang (Zhao et al. 2021), *Aspleniumdanxiaense* K.W.Xu (Xu et al. 2022), *Pileadanxiaensis* L.F.Fu, A.K.Monro & Y.G.Wei (Fu et al. 2022), *Wikstroemiafragrans* W.B.Liao, Q.Fan & J.R.Chen (Chen et al. 2022), and *Commelinadanxiaensis* Q.Fan, Long Y.Wang & W.Guo (Wang et al. 2023). As a World Heritage site and tourist attraction, Danxia landform possesses special and complicated habitat differences at a small scale, which might contribute to the plant endemism at Mount Danxia (Peng et al. 2018).

## ﻿Taxonomic treatment

### ﻿ Sedum jinglanii

Taxon classificationPlantaeSaxifragalesCrassulaceae

Yan S.Huang & Q.Fan sp. nov.

35584965-9C8A-516F-823A-890BDDE1C6C8

urn:lsid:ipni.org:names:77315511-1

#### Type.

**Figure 2.**
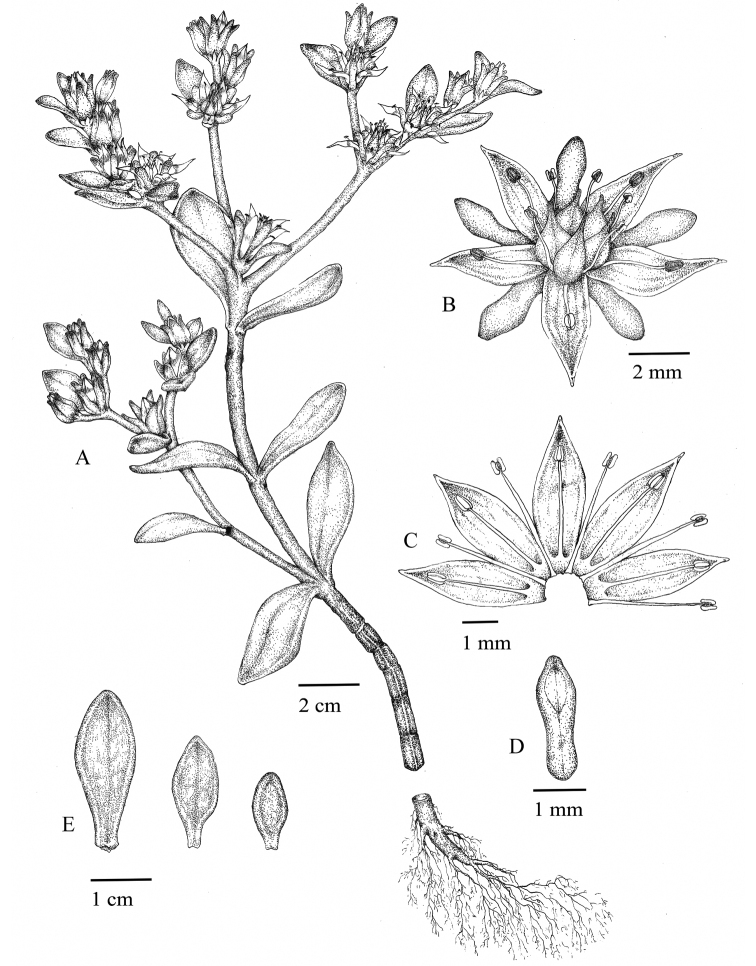
*Sedumjinglanii* sp. nov. **A** habit **B** flower with sepals, petals, stamens and carpels **C** petals and stamens **D** sepal **E** leaves. Illustration by Yuan-Yuan Sun based on living field-collected material (*Y. S. Huang 21040301*).

**Figure 3.**
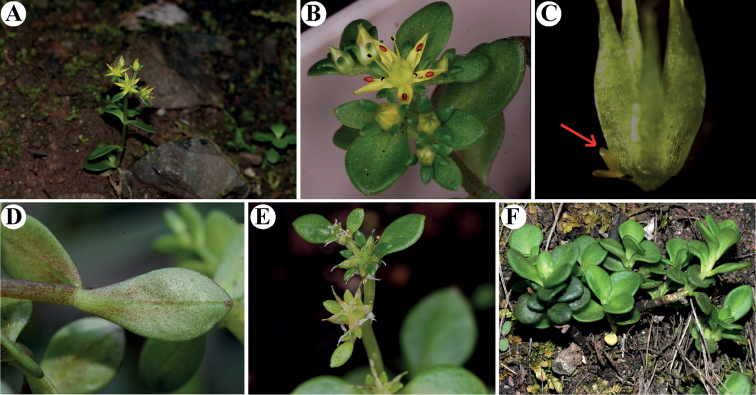
*Sedumjinglanii* sp. nov. **A** habit **B** flower, front view, showing brownish-red anthers **C** carpels and a nectar scale (red arrow) **D** abaxial leaf surface **E** young unripe fruits **F** young sterile individuals **A, B, D** photographed by Qiang Fan in the field (Pingtouzhai, 6 April 2022, *Q. Fan et al.*, *DNPC 1953*) **C** photographed by Min Lin in the lab (7 April 2022, *Q. Fan et al.*, *DNPC 1953*) **E, F** photographed by Yan-Shuang Huang (Bazhai, 3 April 2021, *Y. S. Huang 21040301*).

China. Guangdong Province, Renhua County, Mount Danxia, Bazhai, in the cliff of steep slopes, 25°00'N, 113°39'E, 520 m a.s.l., 3 April 2021, *Y. S. Huang 21040301* (holotype: SYS; isotype: SYS) (Figs 2, 3).

#### Diagnosis.

This new species is similar to *S.alfredi*, but differs from the latter in its opposite leaves (vs. alternate leaves), its usually wider leaves (0.8–2.9 × 0.4–1.2 cm vs. 1.2–3.0 × 0.2–0.6 cm), usually shorter petals (3.4–4.5 mm vs. 4–6 mm), shorter nectar scales (0.4–0.5 mm vs. ca. 0.5–1 mm), and shorter carpels (1.5–2.6 mm vs. 4–5 mm). Although the leaves of this new species and of *S.emarginatum* are opposite, it can be easily distinguished from the latter by its short, erect or ascending rhizomes (vs. long and prostrate rhizomes), shorter petals (3.4–4.5 mm vs. 6–8 mm) and shorter carpels (1.5–2.6 mm vs. 4–5 mm).

#### Description.

Fleshy herbs, perennial; stems glabrous, greenish, often with small reddish dots thus appearing more or less reddish, ascending; leaves opposite, usually deciduous, crowded distally on the stem, succulent; leaf blade spatulate or obovate, 8–29 mm long, 4–12 mm wide, base narrowly cuneate and spurred, apex obtuse and sometimes emarginate; inflorescence in dense terminal cymes, usually two to four branched; bracts leaflike, 1.7–2.4 mm long, 0.7–1.1 mm wide; flowers usually sessile, rarely with short pedicels to 0.8 mm long, unequally 5-merous; sepals green, linear-spatulate, 2–3.1 mm × 0.7–1.4 mm, base shortly spurred; petals yellow, lanceolate to lanceolate-oblong, 3.4–4.5 mm × 0.8–1.1 mm, base connate for 0.1–0.2 mm, apex mucronate; stamens 10, yellow, filiform, arranged in 2 whorls; antesepalous ones 3.2–3.3 mm, antepetalous ones 2.2–2.6 mm; anthers brownish red, long ellipsoid. Nectar scales yellow green, spatulate-quadrangular, 0.4–0.5 × 0.2–0.3 mm, apex obtusely truncate. Carpels yellow green, erect, ovoid-lanceolate, 1.5–2.6 mm long, 0.6–0.9 mm wide, adaxially gibbous, base shortly connate; styles 0.6–0.9 mm long. Follicles yellowish, obliquely divergent. Seeds numerous, brown, oblong, 0.5–0.6 mm, papillate.

#### Phenology.

Flowering from April to May. Fruiting from June to August.

#### Etymology.

*Sedumjinglanii* is named after Prof. Jing-Lan Feng (1898–1976), an academician of the Chinese Academy of Sciences and one of the founders of mineralogy in China. In 1928, he discovered and named the red beds and related strata in North Guangdong as “Danxia Formation” for the first time (Peng 2020).

#### Distribution and habitat.

Presently, this new species is only known from the type locality, Mount Danxia, Renhua County, Guangdong Province, China. It grows on the cliff of steep slopes at altitudes of 200–550 m a.s.l.

#### Conservation status.

Only five populations were found with no more than 1,000 mature individuals. Thus, the conservation status could be considered as Vulnerable (VU; D1), according to the IUCN Red List Criteria (IUCN Standards and Petitions Subcommittee 2022).

#### Additional specimens examined

**(paratypes).** China. Guangdong: Renhua County, Mount Danxia, Pingtouzhai, 25°00'N, 113°37'E, 536 m a.s.l., 6 April 2022, *Q. Fan et al.*, *DNPC 1953* (SYS); Renhua County, Mount Danxia, Yanyan, 25°02'N, 113°61'E, 263 m a.s.l., 27 December 2022, *Q. Fan et al. DNPC 2873* (SYS).

## Supplementary Material

XML Treatment for Sedum jinglanii
